# Ultrasound-Assisted Production of Xylo-Oligosaccharides From Alkali-Solubilized Corncob Bran Using *Penicillium janthinellum* XAF01 Acidic Xylanase

**DOI:** 10.3389/fbioe.2021.755003

**Published:** 2021-09-10

**Authors:** Mingchun Zhou, Guangsen Fan, Hanshuo Xia, Xiaohan Zhang, Chao Teng, Xiuting Li

**Affiliations:** ^1^ Beijing Engineering and Technology Research Center of Food Additives, Beijing Technology and Business University, Beijing, China; ^2^ Beijing Advanced Innovation Center for Food Nutrition and Human Health, Beijing Technology and Business University (BTBU), Beijing, China; ^3^ School of Food and Health, Beijing Technology and Business University, Beijing, China; ^4^ College of Food Science and Engineering, Jilin Agricultural University, Changchun, China

**Keywords:** acidic xylanase, corncob, sonication, *Penicillium janthinellum*, xylo-oligosaccharides

## Abstract

A novel treatment involving enzymatic hydrolysis using an acidic xylanase coupled with ultrasound was performed to improve the xylo-oligosaccharides (XOS) yield from corncob bran. The acidic xylanase (XynB) was purified to a most suitable pH, temperature, and operational parameters for ultrasound-assisted hydrolysis were determined. A preliminary mechanistic investigation was performed through circular dichroism (CD) spectroscopy, scanning electron microscope (SEM) and a laser particle size analyzer, and the effects of ultrasound on enzyme (XynB) and substrate (corncob bran) were assessed. The results show that the maximum XOS yield was 20.71% when the reaction pH and temperature were 4.3 and 50°C, the ultrasonic parameters were 50 kHz and 0.40 W/cm^2^, which was 2.55 fold higher than that obtained using a non-ultrasound-assisted enzymatic preparation. Mechanism studies indicated that ultrasonic pretreatment could reduce the β-fold content and increase the random coil content. Changes in structure and size of substrate were observed. The specific surface area of the XAC molecules is easy to carry out enzymatic reaction, which is beneficial to the production of XOS.

## Introduction

Lignocellulosic materials are the most abundant organic residues worldwide. Grains including wheat straw, rice straw, corn cobs and tobacco straw are rich in lignocellulose and are potential industrial feeds (Chapla et al., 2012). Plant biomass is an economical, available, and renewable source of biofuel, bioenergy, and a variety of value-added biomolecules (Kumar and Satyanarayana, 2015). Corncob contains about 35% xylan, which is an essential by-product of that industry. It has many functions and can be used as animal feed or return to harvested farmland (Aachary and Prapulla, 2009). It is noteworthy that the xylan, a complex five-carbon polysaccharide, is the main component in hemicellulose (Liu et al., 2021). The xylan-rich lignocellulosic materials contain a large amount of xylan, which are composed of two to seven xylose units connected by β-1,4-glycosidic links (de Figueiredo et al., 2017; Freitas et al., 2019), selectively increase the growth of Bifidobacteria and *Lactobacillus* and exhibit antioxidant activities (Yamani et al., 2016; Zhang et al., 2018; Khangwal and Shukla, 2019). In addition, research on XOS has proven that it can be widely used in diet, health care industries, animal’s husbandry, chemical and pharmaceutical industries (Moniz et al., 2016; Khangwal et al., 2020). The general strategies used for XOS production include pretreatments and hydrolysis (Kumar and Satyanarayana, 2015; Freitas et al., 2021). Hemicellulose extraction involves various pretreatments, which ease the subsequent XOS producing steps. At present, the main methods for pretreatment include: heat-dilute-acide hydrolysis (Liu et al., 2012), organic solvent extraction (Zhang et al., 2016), pyrohydrolysis of hemicellulose (Zhao et al., 2019) and alkaline extraction (Rashid et al., 2020). Compared to other pretreatment methods, alkaline extraction operating conditions are less demanding (lower temperature and pressure) (Badiei et al., 2014) and promote the dissolution of lignin and also protect cellulose from drastic degradation (Liu et al., 2021). It is considered an effective way to pursue high xylan extraction rates, low cost and high purity. In fact, alkaline hydrolysis causes the xylan to be subjected to endo-xylanases, resulting in high yield of XOS (Akpinar et al., 2010).

Ultrasound, a green method of extracting natural products, is a new concept that is both environment and user-friendly; at the same time, it can enhance the competitiveness of the industry and make the technology more environmentally friendly, economical and innovative (Chemat et al., 2017). This emerging technology has been used as an alternative to conventional food processing operations for inactivating or accelerating enzymatic activity and facilitating the extraction of various bioactive components (Gallego-Juárez et al., 2010). Although some studies have reported that a combination of ultrasound and pectinase (Larsen et al., 2021), cellulase (Szabo and Csiszar, 2017), lipase (Jadhav et al., 2021), alpha-amylase (Gaquere-Parker et al., 2018), alkaline protease (Pawar and Rathod, 2018) and saccharification enzyme (Wang et al., 2017) accelerates the enzymatic reaction. The results may be due to effects on enzyme behavior, changes in substrate structure, and effect of the reaction medium (Jian et al., 2008). Yet, reports on the hydrolysis of xylan catalyzation of ultrasound-assisted xylanase can be rarely seen. Long-term exposure to high-intensity ultrasound can restrict the enzymes’ catalytic activity. However, in certain cases, enzyme activities have increased following short exposures to ultrasound (Joshi and Gogate, 2019; Farzadkia et al., 2021).

Many microorganisms are the origin of xylanase producers and there already are considerable documents on xylanase in fungi which are intensively studied (Uma et al., 2016). Studies have reported the opinion that xylanases have optimal activity at mesophilic temperatures and moderate or slightly acidic pH. Contrary to alkalophilic xylanases (active at pH ≥ 8.0), only few of their acidophilic counterparts (active pH between 1.0 and 5.0) have been studied so far (Kallel et al., 2016). The objective of this study was to evaluate the effect of acidic xylanase (from *Penicillium janthinellum* XAF01) on XOS production in the presence of low intensity ultrasound treatment. The circular dichroism (CD) spectra of xylanase and the scanning electron micrographs of xylan were also analyzed for determining the mechanism underlying ultrasound treatment.

## Materials and Methods

### Raw Material and Preparation of Hemicellulosic From Corncob

The raw material corncob used in this work was provided by a XOS factory (Shandong Longlive Biotechnology Co.) in Shandong province, China. Dry in an oven at 60°C for 24 h, mill (60–80 mesh), and store in a closed polycarbonate container. The composition of the corncob was 45.7% cellulose, 33.3% hemicelluloses, 18.0% lignin, and 3.0% ash on a dry weight basis. Xylan was prepared according to the method of Longue Júnior with some modifications (Longue Junior et al., 2013). The optimal process conditions can be got from single factor experiment carried out by using four main factors: corncob concentration, pH, temperature and time: corncob was pretreated with 10% NaOH, with a liquid to solid ratios (L:S) (mL:g) of 10:1, at 100°C for 2 h. The liquid fraction was collected, neutralized to pH 7.0 with HCl, concentrated, and then freeze dried. The yield of the hemicellulosic fraction was 22.7% (hemicellulosic fraction/g corncob). Corncob xylan extracted using NaOH was abbreviated as XAC, which was then used as the substrate for XOS production. All other chemicals were analytical grade and commercially available unless otherwise stated.

### Strain and Culture Conditions

*P. janthinellum* XAF01 was isolated from soil samples collected from Honghe, Yunnan province, China. For the production of xylanase, *P. janthinellum* XAF01 was cultivated in 250 ml Erlenmeyer flasks containing 60 g/L corncob as carbon source in 50 ml minimal medium (0.2% NaNO_3_, 0.6% KH_2_PO_4_, 0.05% MgSO_4_⋅7H_2_O, 0.15% K_2_HPO_4_) supplemented with 0.3% yeast extract and 1.0% beef peptone. Under a condition of pH 3.5, temperature at 25°C and lasting for 7 days, cultures grew with shaking at 125 r/min. After growth, the culture supernatant was separated from the mycelium by centrifugation at 10,000 r/min for 20 min. The supernatant solutions, hereafter called crude extracts, were stored at 4°C for subsequent use.

### Enzyme Assay and Purification

#### Enzyme Assay

Endo-β-D-xylanase (EC3.2.1.8) activity was determined using the modified dinitrosalicylic acid (DNS) method (Bailey et al., 1992). Briefly, 100 μL of the culture supernatant solution and 900 μL of a 1% (w/v) beechwood xylan (Sigma-Aldrich Pvt. Ltd., United States) suspension in 50 mM citrate buffer (pH 4.0) were mixed. The incubation of mixture was carried out at 55°C for 5 min and with the addition of 1000 μL DNS, the reaction can be stopped. The xylanase activity unit is the needed amount of enzyme to release 1 μmol xylose equivalent in every 60 s.

Protein concentrations were determined using Lowry’s method (Lowry et al., 1951), and BSA (bovine serum albumin) used as the standard was purchased from Roche (738328).

#### Enzyme Purification

Each purification process was executed at 4°C. The crude enzyme was first purified with 20–50% ammonium sulfate (Sinopharm Chemical Reagent CO., Ltd.); the preliminary purified enzyme solution was subjected to dialysis for 12 h with 20 mM sodium acetate buffer, pH 3.8, and then loaded on a SP-Sepharose Fast Flow ion-exchange column equilibrated with 20 mM sodium acetate buffer, pH 3.8. The elution was carried out with a linear NaCl gradient (0–100 mm in 20 mM sodium acetate buffer, pH 3.8) at a flow rate of 1.0 ml/min.

All active fractions were concentrated to 1.0 ml and slowly loaded on a Q-Sepharose Fast Flow column, which was equilibrated with 20 mM citrate buffer (pH 5.6). At a flow rate of 1.0 ml/min, 2 ml fractions were collected after eluting protein. Fractions exhibiting xylanase activity were then collected for further use.

#### Sodium Dodecyl Sulfate-Polyacrylamide Gel Electrophoresis and Zymogram

Enzyme purity and molecular weight can be determined by SDS-PAGE and 12.5% (w/v) separation and 4.5% (w/v) stacked gel (Laemmli, 1970). By Coomassie Brilliant Blue G 250, the gel was stained. And the molecular weight of the enzyme is determined with a low molecular weight scale label.

The xylanase activity was detected by incubation with a zymogram gel containing 1% (w/v) birchwood xylan (Sigma-Aldrich Pvt. Ltd., United States) at 40°C for 2 h, followed by staining in Congo red solution (1 mg/ml). The dye was removed using 1 M NaCl solution till clear areas in a dark red background appeared, which is indicative of xylanase activity (Schwarz et al., 1987).

### Biochemical Characterization of the Purified Xylanases

#### Effect of pH on Xylanase Activity and Stability

With 1% beechwood xylan as the substrate, the best pH value required in xylanase activity can be determined at 55°C in the pH range of 2.70–9.00. The highest xylanase activity was used to define 100% activity. The following buffer systems (50 mM) were used: citrate buffer, pH 2.70–5.70; acetate buffer, pH 3.20–5.80; MES buffer, pH 5.20–7.20; MOPS buffer, pH 6.20–8.20; Tris-HCl buffer, pH 7.00–9.00.

The effect of pH on the stability of xylanase was evaluated by incubating the enzyme at different pH values for 30 min at 50°C. Samples were then cooled with ice water. Afterwards, residual activity of each sample was determined under standard conditions.

#### Effect of Temperature on Xylanase Activity and Stability

The optimal temperature required for enzyme activity was determined in the range of 40–60°C. In each case, samples were diluted in the optimal pH buffer solution prior to xylanase activity assay. The thermal stability of the immobilized and free xylanase were determined by incubating the enzyme in the optimal pH buffer solution for 5 h at different temperatures (40–60°C). At certain time intervals, aliquots could be extracted, and under standard conditions, residual activity can be tested. The 100% activity can be determined by the unheated enzyme.

#### Ultrasound-Assisted Hydrolysis of Hemicellulosic Fraction

Ultrasonic hydrolysis was carried out using a DTD5200S ultrasonic device (Beijing Hongxianglong Biotechnology Co., Ltd.) with an ultrasonic power of up to 300 W, a frequency of 135 kHz, and a microtip diameter of 10 mm. The instrument used was a US bath type reactor with five different 2.0 cm ultrasound generator probes at four different frequencies (28, 40, 50, and 128 kHz); all probes can deliver maximum power of 300 W. Prior to hydrolysis, XAC was powdered into 0.45 mm particles. Subsequently, the ground XAC were dispersed at liquid to solid ratios of 50:1 (mL:g), in 10 ml 50 mM acetate buffer (pH 3.7) and the reaction was performed using 16U/mL acidic xylanase at 40°C for 40min. The prepared XAC was completely mixed in a beaker 3 cm shorter than the water bath, and the probe of the ultrasonic generator (about 1 cm to the liquid level) was inserted into the ultrasonic field immediately. The ultrasound intensity released from the probe was regulated to 0.04, 0.13, 0.22, 0.31, 0.40 W/cm^2^ and the ultrasound frequencies was regulated to 28, 40, 50, and 128 kHz. Each treatment was replicated thrice.

#### Determination of the Kinetic Parameters of the Michaelis–Menten

The function of ultrasound (0.31 W/cm^2^ and 50 kHz) on acidic xylanase was evaluated. The enzyme activity of xylanase at 40°C for 5 min was determined by different substrate concentrations, and the kinetic parameters of xylanase on beech xylan were determined; with the help of “GraFit” software, we calculated *Km* and *Vmax* values.

### Test of Effect of Ultrasound on Xylanase XynB and the Substrate

#### CD Measurement of Acidic Xylanase XynB

Enzyme XynB (0.20 mg/ml) in 0.05 M acetate buffer (pH 3.8) was sonicated at 40°C using three categories or protocols for monitoring changes in enzyme structure under different ultrasound conditions. The first group of experiments studied the effect of ultrasound with different sound intensity (0.04, 0.13, 0.22, 0.31, 0.40 W/cm^2^) at 50 kHz for 40 min. The second set of experiments was performed at different ultrasonic frequency (28, 40, 50, 128 kHz) at 0.31 W/cm^2^ for 40min. The third set of experiments was performed at different time points (10, 20, 30, and 40 min) at 0.31 W/cm^2^ and 50 kHz.

After ultrasound, CD spectra were recorded by spectrometer (JASCO, Tokyo, Japan; J-815 type), and CD spectra were recorded by a quartz container with 1 mm optical path length at room temperature (20 ± 1°C). CD spectra were scanned in the far ultraviolet (260–190 nm) range with a repetition rate of 100 nm/min and a bandwidth of 0.1 nm. CD data are represented by molar ellipticity in mde. cm^2^. dmol^−1^. The α-helix in the measured molar ellipticity of xylanase was observed at 208 nm (Wang et al., 2003).

#### Characterization of XAC Particles

The particle sizes of xylan (XAC) and its aggregates in XAC suspension and acoustic XAC suspension were measured by TopSizer laser diffraction particle size analyzer (OMEC, Zhuhai, China). Which can provide useful information for comparing the size of non-spherical rod like xylan whiskers and their aggregates before and after sonication.

#### Scanning Electron Microscopy

The morphology of XAC particles as well as ultrasonic pretreated samples was observed with a VEGA\\LSU scanning electron microscope (Tescan Company, Czech Republic) at 15 kV accelerating voltage. The samples were covered with a thin layer of gold as a conductive medium under a scanning electron microscope.

### Hydrolysate Analysis Using High Performance Liquid Chromatography

The degradation products in the hydrolyzate were quantitatively analyzed by effective solution chromatography (HPLC, Agilent 1260 series, Agilent Technologies, United States) equipped with a refractive index detector (Amel et al., 2016). The separation was achieved with InterWAX (KS-802) column (DM) and a RID-10 A refractive index detector. All samples were filtered through a 0.22 mm filter prior to measurement. The column was kept at 80°C and washed with high performance liquid chromatography on water at 0.6 ml/min flow rate. The concentration of oligosaccharides was determined by peak area method and compared with X2, X3 and oligosaccharides purchased from Megazyme (Ireland). The XOS yield (w/w) was worked out to be (X2 + X3)/xylan weight.

### Statistical Analysis

At the significance level of *p* < 0.05, the effect of ultrasound could be comparable by variance analysis (ANOVA). OriginPro 8.0 was used for all graphs and calculations.

## Results and Discussion

### Purification and Biochemical of Xylanase XynB

**FIGURE 1:**
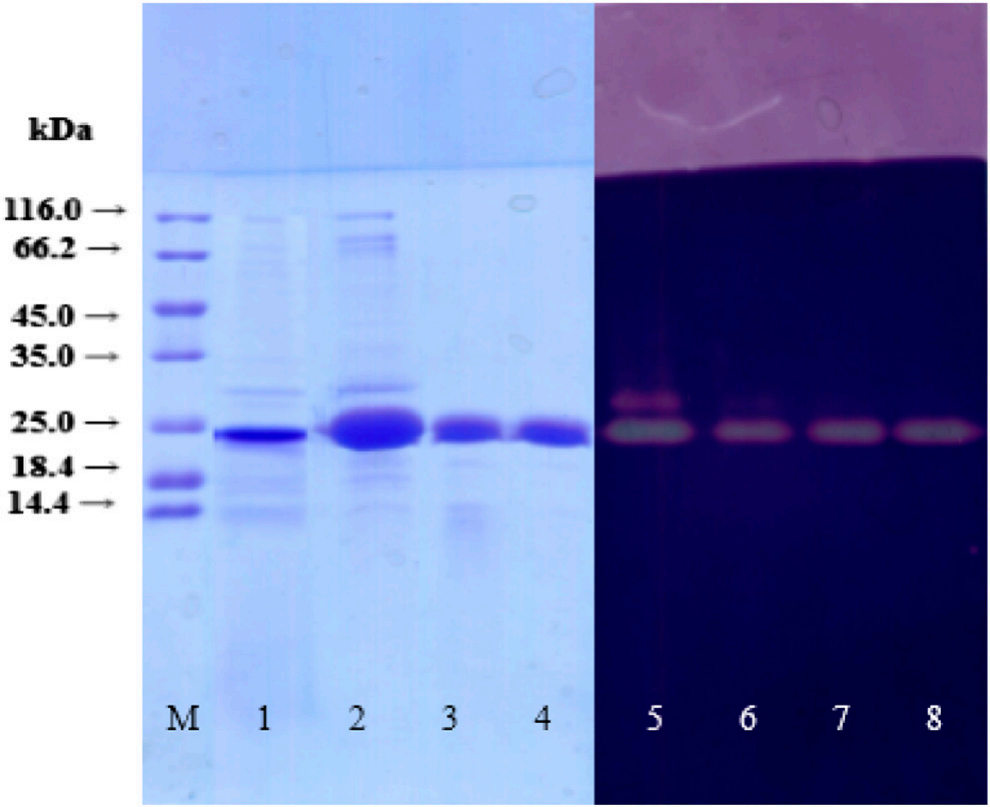
SDS-PAGE and zymogram of XynB during purification. Lane M, SM031 standard protein marker (14.4–116.0 kDa); lane 1, crude extract; lane 2, 50–70% ammonium sulfate precipitation; lane 3, a fraction of SP-Sepharose Fast Flow purification; lane 4, a fraction of Q-Sepharose Fast Flow purification; lane 5, zymography of crude extract; lane 6, zymography of 50–70% ammonium sulfate precipitation; lane 7, zymography of fractions of SP-Sepharose Fast Flow purification; lane 8, zymography of purified xylanase of Q-Sepharose Fast Flow purification.

The xylanase XynB-producing strain *P. janthinellium* XAF01 was evaluated using liquid-state fermentation. Maximum xylanase activity of 1807.9U/mL was observed in the presence of 6% corncob and 1.5% ammonium sulfate as the most appropriate inorganic nitrogen source. XynB from *P. janthinellum* XAF01 was purified using a three-step procedure described in 2.3.2. After the final step, the purification ratio of the enzyme was 1.70 times, the recovery was 2.09%, and the specific activity was 540.4 U/mg protein (Table 1). The purification of the final eluted protein was determined by SDS-PAGE and its molecular weight was estimated to be 24.1 kDa (Figure 1). The xylanase activity of purified XynB was determined by zymography.

**TABLE 1:** Summary of xylanase purification from *Penicillium janthinellum* XAF01.

Purification step	Total activity(U)	Total protein (mg)	Specific activity (U/mg)	Purification factor (-fold)	Yield (%)
Crude enzyme extracts	36182.00	113.90	317.66	1.00	100.00
50–70% ammonium sulfate salt out	25564.90	10.80	2367.12	7.45	70.66
SP-Sepharose fast flow	1055.90	2.30	459.09	1.45	2.92
Q-Sepharose fast flow	756.60	1.40	540.43	1.70	2.09

**FIGURE 2:**
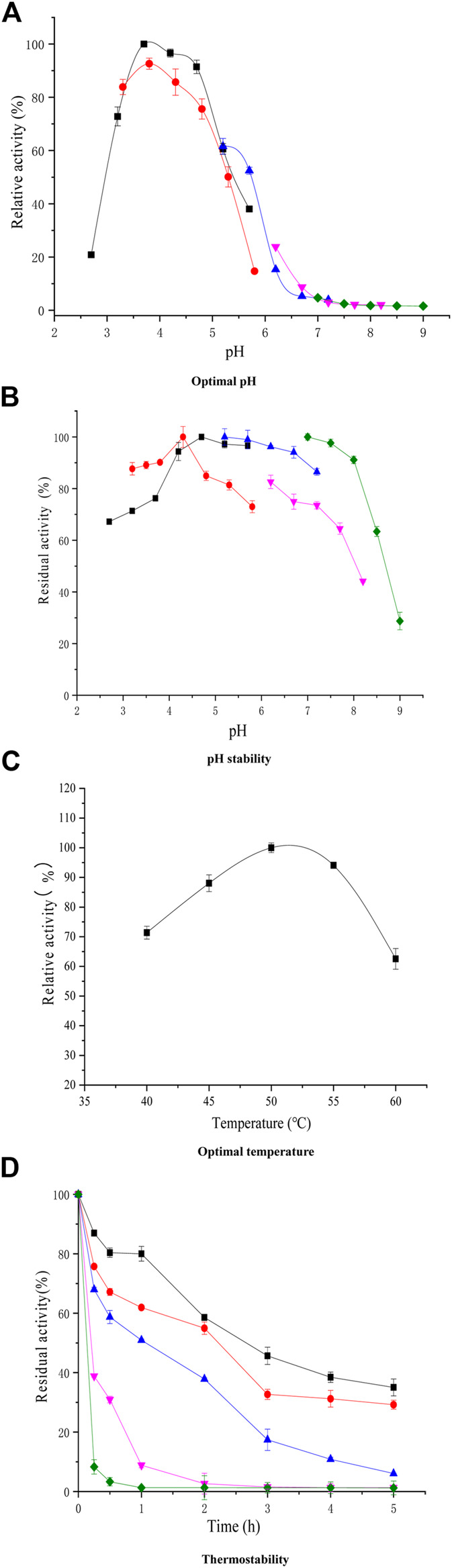
Temperature effect [**(C)**; pH 3.8] and pH on xylanase activity [**(A)**; 55°C] of purified XynB and thermal stability [**(D)**; 40–60°C] and pH stability [**(B)**; pH 2.70–9.00] test for XynB. (■, Citric acid; ●, Acetic acid; ▲, MES; ▼, MOPS; ♦, Tris + HCL. Symbols for thermal inactivation: (■, 40°C; ●, 45°C; ▲, 50°C; ▼, 55°C; ♦, 60°C).

The purified xylanase XynB was most active at pH 4.3 and 50°C (Figures 2B,C), The optimal pH and temperature required for XynB activity were mostly similar to that of purified GH11 xylanase from *Aspergillus kawachii* and *Streptomyces actuosus*, which presented highest activity at pH 4.5 and 4.0 (Dalagnol et al., 2017; Wang et al., 2017). Furthermore, XynB was stable in the pH range of 4.0–8.0, with residual activities >80% after treatment for 0.5 h (Figure 2B). In addition, Figures 2 C,D show that XynB was stable at 40°C and 50°C, and the residual xylanase activity was >60% when incubated for 2 h at 45°C.

### Effect of Low Intensity Ultrasonic Pretreatment on XAC Hydrolysis

**FIGURE 3:**
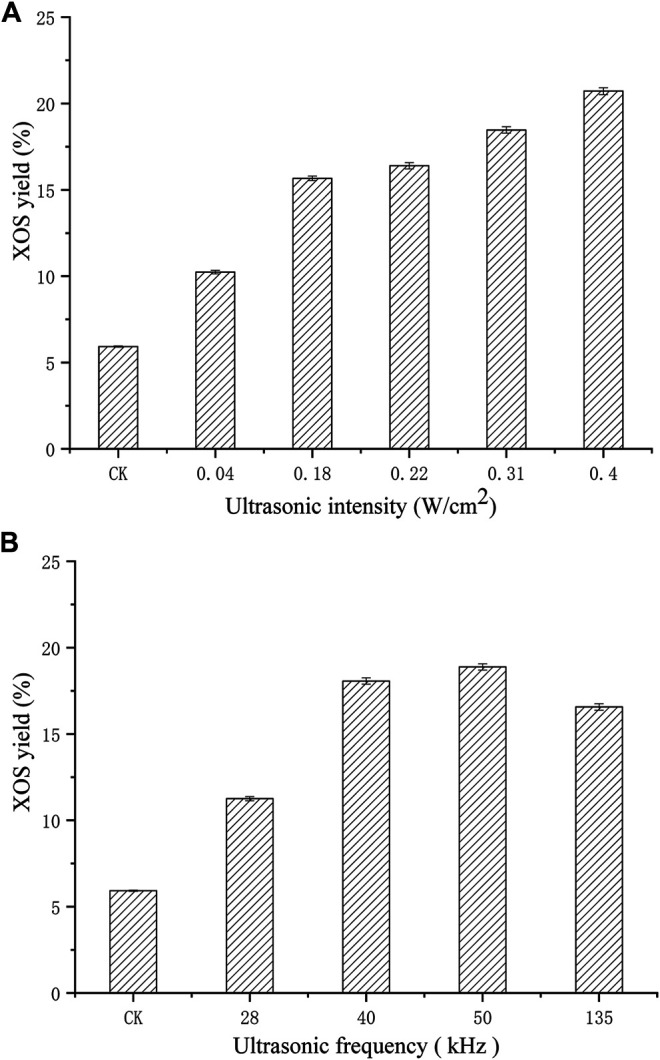
Effects of pretreating XynB with different ultrasonic intensity **(A)** on xylanase hydrolysis at 50 kHz for 40 min, and ultrasonic frequency **(B)** on xylanase hydrolysis at 0.31 W/cm^2^ for 40 min.

Figure 3 illustrates the effect of low intensity ultrasonic treatment on XOS yield. Under the same experimental conditions, the yield of XOS without ultrasonic treatment was used as a control, the results show that extended processing time (40 min) can significantly improve XOS yield. Ultrasound intensity is listed as one of the key parameters affecting ultrasonic cavitation. In general, cavitation effect increases with ultrasonic power, and the increase continued till ultrasonic power reached 0.40 W/cm^2^, and the XOS yield was 20.71%, which was 2.55 fold higher than that of the control (the yield of XOS without ultrasonic treatment was 5.92%). This is probably because ultrasound affects the xylan or xylanase molecular bonds directly (the deduction was confirmed by further experiments discussed later), and so does ultrasonic pretreatment frequency. Ultrasonic frequency range of 28–135 kHz corresponds to ultrasonic wavelength of 50, 35, 28, and 10.37 mm separately, which is larger than the size of xylanase and xylan molecules. The XOS yield increased considerably at all frequencies, and maximum XOS was obtained when the pretreatment frequency was 50kHz, with an abrupt decrease at 135 kHz. This is the first study to show ultrasound-assisted xylanase-catalyzed hydrolysis of xylan and hence the presented results are novel.

Fundamentally, the main mechanisms for the increase in XOS yield are related to the micro-jet, flow field, turbulence and shear forces generated during the sonar process. Although several published studies showed that pretreatment of xylan was necessary prior to enzymatic hydrolysis, particle size and solubility was believed to affect hydrolysis of carbohydrates with high molecular weight (Wang et al., 2017).

Sonication pretreatment of enzyme (XynB) and substrates (XAC) was performed separately prior to enzymatic hydrolysis, which improved XOS recovery, albeit not significantly, compared to direct hydrolysis. Hence, superposition of effect enhancement may occur during ultrasound-assisted enzymatic hydrolysis. Ultrasound may be used to modify the kinetics of enzyme catalysed reactions. Mass transfer can be enhanced by strong shear and microfluidics associated with cavitation as they may alter the xylan chain, making xylanolytic enzymes more accessible to the substrate (Dalagnol et al., 2017). This is consistent with the results of ultrasound-assisted starch hydrolysis studies (Wang et al., 2017). Ultrasound-mediated improvement in enzymatic reactions can be explained using the following three parameters: substrate, enzyme, or combination of both substrate and enzyme. The effect of any of these parameters may vary with substrate characteristics (particle size, solubility), enzyme characteristics, and reaction conditions. Studies showed that ultrasound can even strengthen the binding of enzymes to substrates and the removal of products from enzymes in reactions (Dalagnol et al., 2017).

### Ultrasonic Treatment

#### Effect of Ultrasonic Treatment on the Kinetic Parameters

By nonlinearly fitting the kinetic data to the Michaelis-Menten equation, the kinetic parameters, maximum reaction rate (*V*_max_), Michaelis constant (*K*_*M*_), and catalytic efficiency (*V*_max_ */K*_*M*_) can be calculated. The *V*_max_, *K*_*M*_, and *V*_max_/*K*_*M*_ of XynB were 189.20 ± 6.15 mg ml^−1^ min^−1^, 23.12 ± 0.98 mg ml^−1^, and 8.18 ± 0.35 min^−1^, respectively. Sonication increased the *V*_max_ and *V*_max_ */K*_*M*_ by 41.1 and 23.5%, respectively. The combination of XynB and ultrasound improved the catalytic ability. *V*_max_ shows the extreme rate of the enzyme reaction when the substrate is saturated, while the *K*_*M*_ value shows the enzyme affinity for substrate. The increase in *V*_max_ indicated strengthening of the xylan-xylanase complex interaction, and accelerated hydrolysis, whereas a slight decrease in *K*_*M*_ indicated an enhancement in the affinity between XynB and XAC, possibly because of better exposure of the xylanase active site after ultrasonication (Dalagnol et al., 2017). That is, ultrasound can increase the rate of reaction and enzyme substrate affinity, and enhance the reaction by enhancing the binding of the enzyme to the substrate. It is produced by mechanical effects of ultrasonic cavitation, and ultrasound’s capability in increasing the mass transfer rate in enzymatic reactions has been reported to be supported (Sajjadi et al., 2017).

#### Effect of Ultrasonic Treatment on Xylanase Structure (CD Spectra Analysis)

Most enzymes are monomeric spherical proteins whose catalytic activity depends on their natural configuration. Ultrasound may alter the secondary structure of the enzyme, thereby exposing the active site of the enzyme to a better extent (Subhedar and Gogate, 2014). The changes of secondary structure of proteins can be analyzed by CD spectroscopy, which calculates the α-helix, β-strand (β-Sheet and β-turn) and random coiling of xylanase, and the relationship between enzyme activity and secondary structure is determined (Li et al., 2015). A typical characteristic of all natural enzyme structure cd spectra is that the XynB spectrum has a fixed minimum peak at 209 nm and a positive peak at 195 nm, which is essentially the same as the native zymogram seen from early researches (You et al., 2010). The characteristics of proteins rich in β-chain (β-Sheet and β-turn) secondary structures can be found in these two spectra. The comparison of secondary structure content obtained using deconvolution of CD revealed an obvious difference regarding β-sheet, β-turn, and random coil. This analysis showed a slight decrease in XynB β-strand content and increase in random coil content with increase in ultrasonic intensity (from 0.04 W/cm^2^ to 0.40 W/cm^2^), which was believed to be related to acceleration of enzymatic hydrolysis. Similar results were obtained when ultrasonic frequency and treatment time were altered (Data was not shown). These results indicated that ultrasonic pretreatment cast no effects on natural secondary structure of XynB, while destroying the hydrophobic interaction between protein molecules, making more internal hydrophobic groups and regions exposed to external environment.

#### Effect of Ultrasonic Treatment on Substrate Particle Size and Microstructure (Characterization of XAC Particles and SEM Analysis)

**FIGURE 4:**
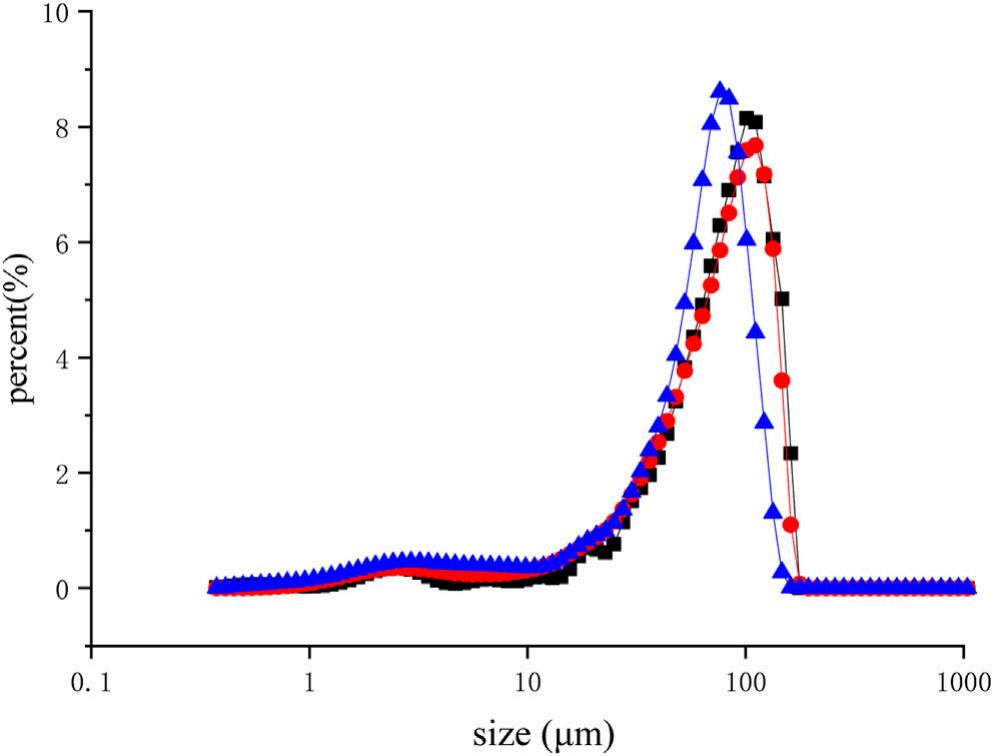
Laser diffraction analysis of aqueous suspensions of XAC using ultrasound (40 kHz, 0.40 W/cm^2^) for different durations. (■, Control; ●, 10 min; ▲, 40 min)

The reduction in size is due to the high shear forces caused by ultrasonic cavitation in liquid mediums (Trujillo and Knoerzer, 2011). After dialysis, the same volume of XAC suspension was sonicated at different times (0–40 min) and then characterized by laser diffraction. A comparison of the particle size distribution of the ultrasonic suspension (Figure 4) shows that the longer the ultrasonic action time, the lower the peak position of the volume distribution (76.43 μm). When the nanoparticles appeared at 40 min (from 84.79 to 65.04 μm), they expanded to smaller particles (Alvira et al., 2010).

**FIGURE 5:**
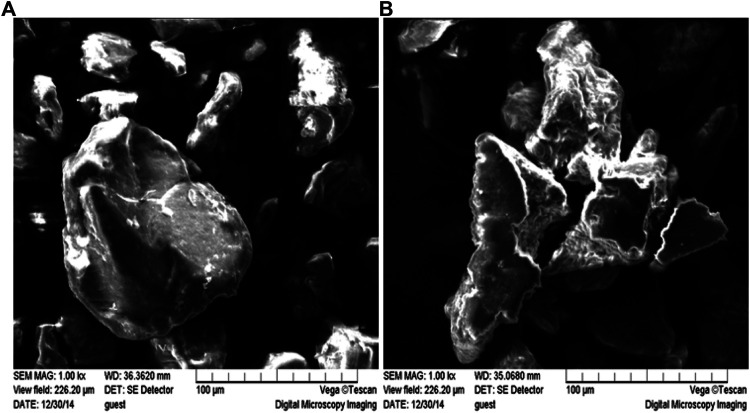
Effects of ultrasound pretreatment of different durations on the microstructure of XAC. **(A)** Original (0min) non-sonicated XAC; **(B)** Ultrasound pretreated XAC for 40 min (50 kHz, 0.40 W/cm^2^).

The microstructure of XAC prior to ultrasound pretreatment was observed using scanning electron microscopy (SEM) as shown in Figure 5. After ultrasonic pretreatment, the XAC structure was loosened and more disordered structures and irregular fragments appeared, which changed the original (0 min) unvoiced XAC suspension with large area and high density. In particular, after long-term ultrasonic pretreatment (40 min), cracks appeared on the surface of XAC; microparticles and small fragments increased the specific surface area of XAC molecules, increasing the probability of contact between the substrate and the enzyme, thereby enhancing the hydrolysis of the enzyme.

Xylan exists in one form and can be linked to other xylan structures and lignin by linking the arabinose-based ferulic acid bridge on the xylan backbone, while the lignin-saccharide complex in the corncob mostly composed of lignin-xylan complex (Van et al., 2011; Ju et al., 2013). Based on the SEM micrographs obtained in this study and calculation of the yield of XOS, we believe that sonication mainly resulted in fiber disruption, which eased subsequent enzymatic hydrolysis. This was in agreement with the observations of Jiang et al. who compared the effects of ultrasound on the functional and structural properties of black-bean protein isolate dispersions (Jiang et al., 2014).

## Conclusion

An acidic xylanase was purified and its properties were studied. Low intensity ultrasound had a pronounce effect on enzymatic hydrolysis of XAC, and increased XOS yield by 61.80% compared to non-pretreated xylanase. The results also showed that separate pretreatment of both xylanase and its substrate accelerated the hydrolysis. Ultrasonic treatment altered the molecular structure and kinetic parameters of xylanase. Evaluation of the effect of pre-sonication of xylan confirmed that the action of ultrasound on the substrate chain destroyed the molecular aggregation and made it easy to carry out enzymatic reaction.

## Data Availability

The original contributions presented in the study are included in the article/Supplementary Material, further inquiries can be directed to the corresponding authors.
